# Supplementary material for: Spinal circuits encode a map of the trunk to control skin twitches
Source: bioRxiv. 2026 May 13:2026.05.10.724081. Preprint. [Version 1] doi: 10.64898/2026.05.10.724081 (PMC13192636; doi:10.64898/2026.05.10.724081)
Supplement: 1 [file NIHPP2026.05.10.724081V1-supplement-1.pdf]

bioRxiv preprint doi: https://doi.org/10.64898/2026.05.10.724081; this version posted May 13, 2026. The copyright holder for this preprint (which was not certified by peer review) is the author/funder, who has granted bioRxiv a license to display the preprint in perpetuity. It is made available under a CC-BY 4.0 International license.

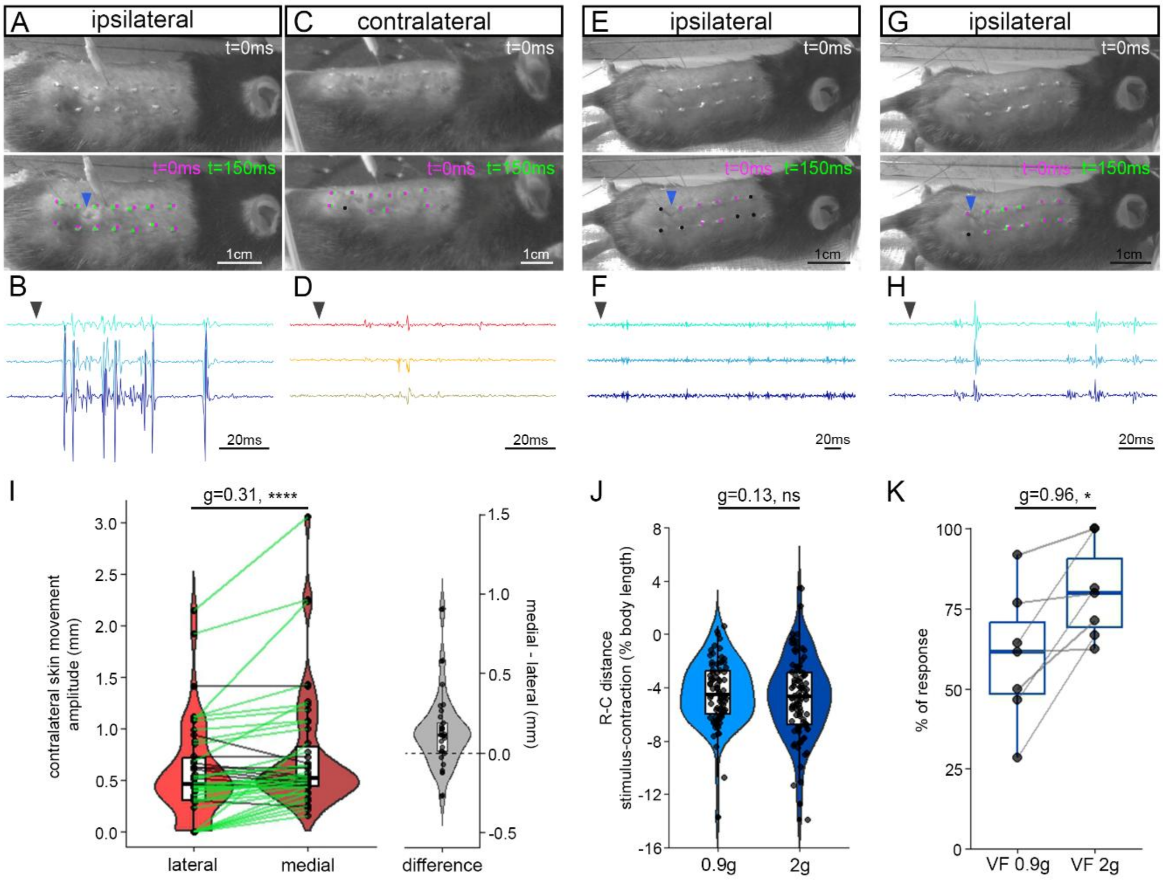

Supplementary figure 1: reflexive skin twitches rely on a spatial representation of the torso. (A, C, E, G) Example of recorded skin movements following stimulation with a 2g pinprick on the side ipsilateral (A) and contralateral (C) to the sensory stimulation. Example of recorded ipsilateral skin movements following stimulation with 0.9g (E) and 2g (G) Von Frey filaments. Images at t=0ms are from the first frame with contact of the pin/Von Frey on the skin, while 150ms later the skin shows a twitch. Magenta and green dots highlight the position of dots placed on the skin to visualize the twitch (magenta for t=0ms, green for t=150ms). Black dots indicate no movement between the two time points. The blue arrowhead points at the position of initial muscle contraction. (B, D, F, H) Differential myomatrix EMG signal recorded from cutaneous maximus from responses showed in the panels above. The black arrowhead indicates the approximate timing of sensory stimulation. (I) Maximum amplitude of skin movement on the side contralateral to the pinprick, on the lines of dots drawn on the lateral versus medial part of the cutaneous maximus (left). Difference of amplitude of contralateral skin movement between the medial and lateral dotted lines (right). Individual trials are connected by green lines when showing a higher value on the medial part of the cutaneous maximus while black lines are used to join trials with higher amplitude on the lateral part of the muscle. Trials from 0.9g and 2g pinprick stimulations are pooled together. (J) Rostro-caudal distance expressed in percent of body length between the sensory stimulation and the location of the initial contraction of the cutaneous maximus following stimulation with a 0.9g or 2g

bioRxiv preprint doi: https://doi.org/10.64898/2026.05.10.724081; this version posted May 13, 2026. The copyright holder for this preprint (which was not certified by peer review) is the author/funder, who has granted bioRxiv a license to display the preprint in perpetuity. It is made available under a CC-BY 4.0 International license.

pinprick. When contraction is caudal to the sensory stimulus the percentage is set as negative, and positive when the shift is rostral. (K) Percentage of trials leading to an ipsilateral skin twitch following stimulation with 2g and 0.9g Von Frey filaments. (I) n = 47 responses (18 from 0.9g and 29 from 2g) in a total of N=8 mice. Non-hierarchically bootstrapped paired Hedge's G coefficients and two-tailed paired Wilcoxon test. (J) n = 92 (2g), and 80 (0.9g) responses in a total of N=5 mice. Non-hierarchically bootstrapped Hedge's G coefficients and two-tailed Mann-Whitney test. (K) Individual values plotted from n=7 mice, two-tailed paired Wilcoxon test and paired bootstrapped Hedge's G coefficients.

bioRxiv preprint doi: https://doi.org/10.64898/2026.05.10.724081; this version posted May 13, 2026. The copyright holder for this preprint (which was not certified by peer review) is the author/funder, who has granted bioRxiv a license to display the preprint in perpetuity. It is made available under a CC-BY 4.0 International license.

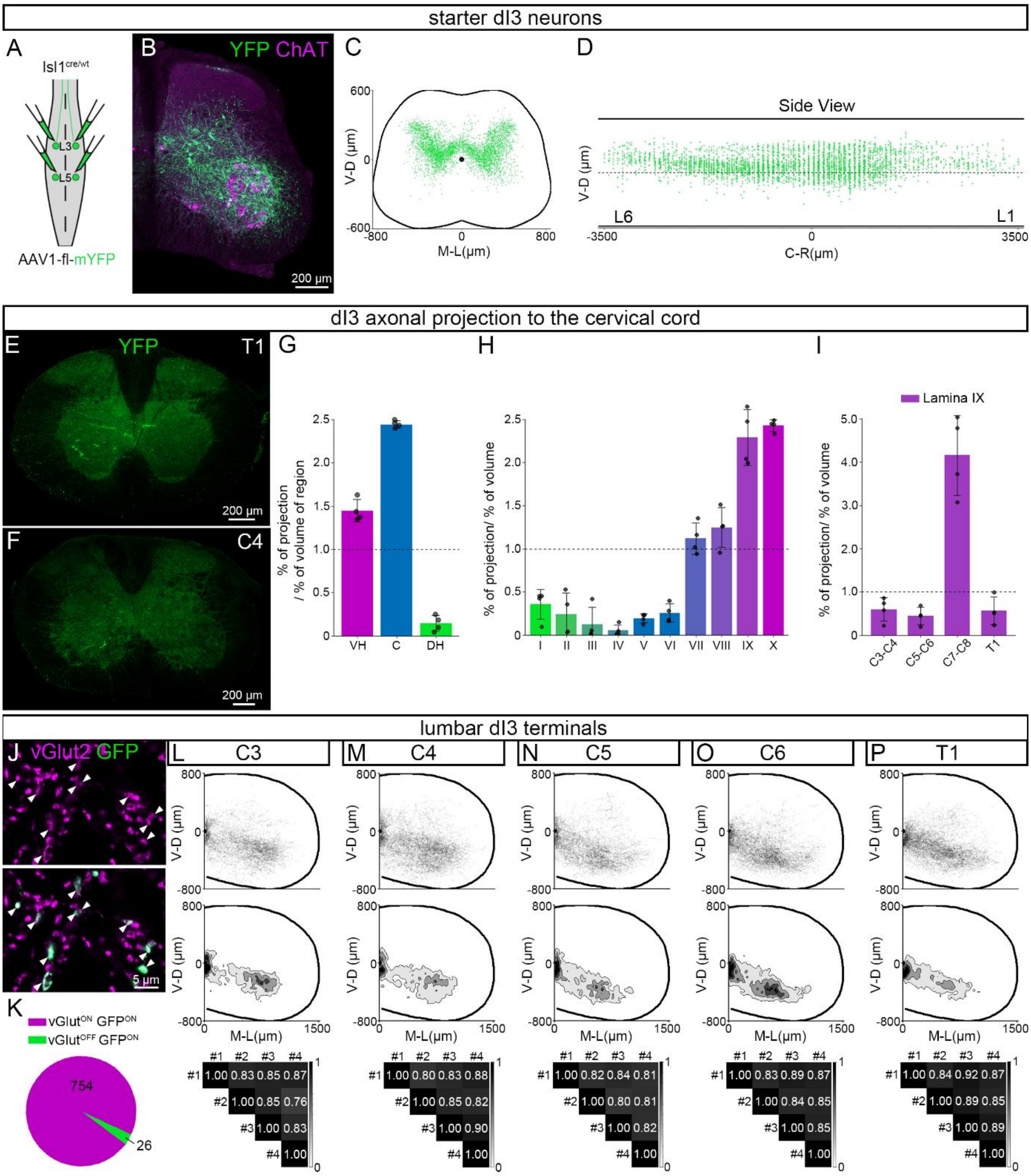

Supplementary figure 2: lumbar dI3 neurons form long ascending projections to cutaneous maximus motor neurons. (A) Experimental strategy used to map axonal projections of lumbar dI3 neurons in cervical segments of the spinal cord. (B) Orthogonal projection showing somata of transduced lumbar dI3 neurons (YFP, green) and cholinergic neurons (ChAT, magenta) on a lumbar cord transverse section. (C-D) Spatial distribution of $YFP^{ON}ChAT^{OFF}$ lumbar dI3 neurons on an idealized lumbar transverse section (C) and sagittal section (D, both sides of the cord overlapped). (E-F) Orthogonal projections showing lumbar dI3 axons (YFP, green) on transverse sections from cervical segment C4 (E) and thoracic segment T1 (F). (G) Percentage of lumbar dI3 neurons axonal projections scaled by the

bioRxiv preprint doi: https://doi.org/10.64898/2026.05.10.724081; this version posted May 13, 2026. The copyright holder for this preprint (which was not certified by peer review) is the author/funder, who has granted bioRxiv a license to display the preprint in perpetuity. It is made available under a CC-BY 4.0 International license.

volume of each region in cervical segments C3 to T1, between ventral horn (VH), central canal area (C) and dorsal horn (DH). (H) Percentage of lumbar dI3 axonal projections in cervical segments (C3 to T1) across spinal cord laminae scaled by their volume. (I) Distribution of lumbar dI3 axonal projections along lamina IX across segments C3 to T1 scaled by its volume. (J) Single optic slice from lumbar dI3 terminals (GFP, green) in lamina IX of caudal cervical segments showing their expression of vGlut2 (magenta). (K) Distribution of lumbar dI3 neurons terminals (GFP^{ON}) colocalized (magenta) or not (green) with vGlut2 signal. (L-P) Spatial distribution, spatial density and spatial correlation analysis of lumbar dI3 terminals in segments C3 (L), C4 (M), C5 (N), C6 (O) and T1 (P) on idealized cervical transverse sections. (C-D, L-P) Spatial distributions and densities are pooled from tracing in n=4 mice. (G-I) Data shown are from n=4 mice, with individual values plotted. (K) Numbers on the pie chart correspond to individual terminals quantified in a total of n=5 mice.

bioRxiv preprint doi: https://doi.org/10.64898/2026.05.10.724081; this version posted May 13, 2026. The copyright holder for this preprint (which was not certified by peer review) is the author/funder, who has granted bioRxiv a license to display the preprint in perpetuity. It is made available under a CC-BY 4.0 International license.

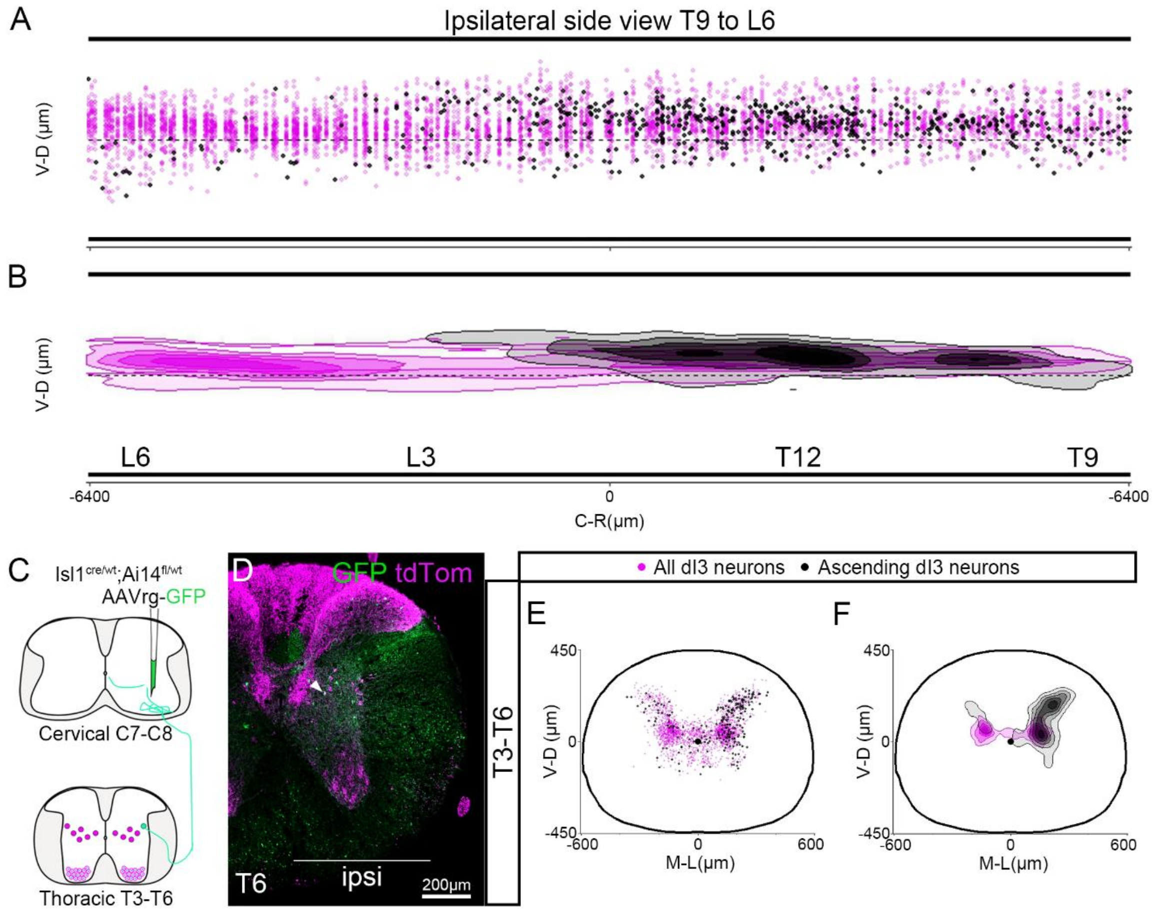

Supplementary figure 3: Long ascending projections are formed by a subset of dorso-lateral dI3 neurons. (A-B) Spatial distribution (A) and density (B) of all ipsilateral dI3 neurons (magenta) and ipsilateral dI3 neurons projecting to the ventral horn of segment C7-C8 (black) on an idealized sagittal section from segments T9 to L6. (C) Experimental strategy used to map on rostral thoracic segments T3 to T6 the global distribution of dI3 neurons (tdTomato^{ON}) and the ones projecting to the ventral horn of C7-C8 segments (GFP^{ON}tdTomato^{ON}). (D) Orthogonal projection of ipsilateral hemislices showing a GFP^{ON}tdTomato^{ON} dI3 neuron (GFP in green, tdTomato in magenta) from thoracic segment T6. (E-F) Spatial distribution (E) and spatial density (F) of all dI3 neurons (magenta) and dI3 neurons projecting to the ventral horn of segment C7-C8 (black) from segments T3 to T6 on an idealized transverse thoracic section. Results pooled from n=4 mice, mapped on every other sections for GFP^{ON}tdTomato^{ON} dI3 neurons while the global distribution of dI3 neurons (tdTomato^{ON}) was mapped on one section every four.

bioRxiv preprint doi: https://doi.org/10.64898/2026.05.10.724081; this version posted May 13, 2026. The copyright holder for this preprint (which was not certified by peer review) is the author/funder, who has granted bioRxiv a license to display the preprint in perpetuity. It is made available under a CC-BY 4.0 International license.

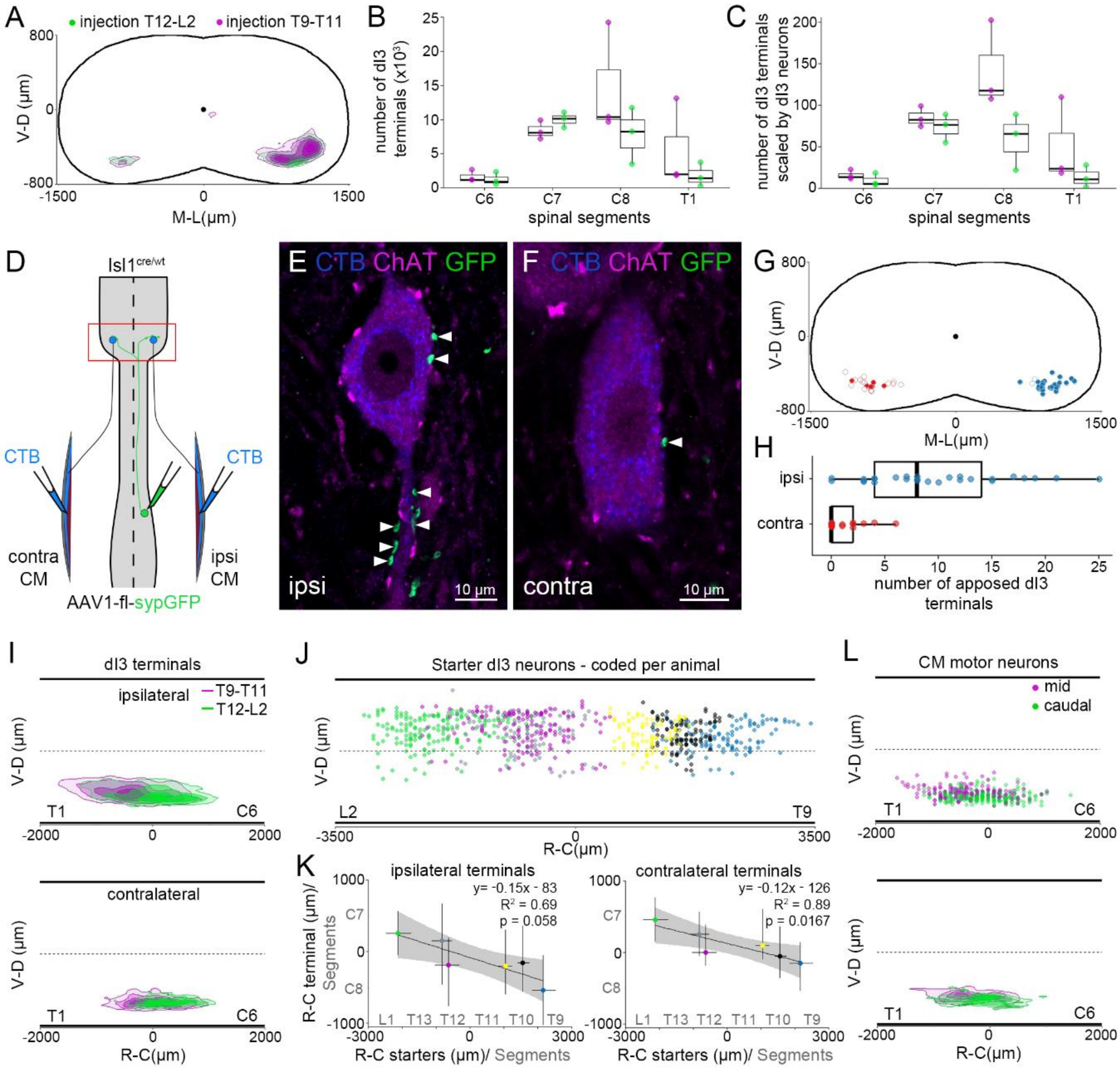

Supplementary figure 4: Cutaneous maximus motor neurons and ascending dI3 projections are somatotopically organized along the rostro-caudal axis. (A) Spatial density of GFP presynaptic terminals from dI3 neurons in T12 to L2 (green) and T9 to T11 (magenta) mapped from segments C6 to T1 on an idealized cervical transverse section. (B-C) Number of dI3 neurons terminals (in thousands, B) and number of dI3 terminals scaled by GFP positive dI3 neurons (C) quantified in every other sections following tracing of dI3 neurons in T9 to T11 (magenta) and T12 to L2 (green). (D) Experimental strategy used to visualize presynaptic terminals from lumbar dI3 neurons on ipsilateral and contralateral identified cutaneous maximus motor neurons. (E-F) Single optic slice showing presynaptic boutons (GFP, green) from dI3 neurons located in segments T12 to L2 on an ipsilateral (E) and a contralateral (F) motor neuron (ChAT, magenta) innervating the cutaneous maximus (CTB, blue). White arrowheads highlight dI3 boutons apposed to the cutaneous maximus motor neurons. (G) Position of analysed ipsilateral (blue) and contralateral (red) cutaneous maximus

bioRxiv preprint doi: https://doi.org/10.64898/2026.05.10.724081; this version posted May 13, 2026. The copyright holder for this preprint (which was not certified by peer review) is the author/funder, who has granted bioRxiv a license to display the preprint in perpetuity. It is made available under a CC-BY 4.0 International license.

motor neurons on an idealized cervical transverse section. Filled circles are motor neurons with apposed terminals from dI3 neurons of segments T12 to L2, while unfilled circles correspond to motor neurons with no apposed terminals. (H) Number of apposed terminals from dI3 neurons in segments T12 to L2 on ipsilateral vs contralateral cutaneous maximus motor neurons. (I) Spatial density of presynaptic terminals from dI3 neurons of T9 to T11 segments (magenta) and T12 to L2 segments (green) along the rostro-caudal axis on an idealized sagittal section from segments C6 to T1. Densities are plotted independently for terminals on the ipsilateral (top) and contralateral (bottom) side to the injection. (J) Spatial distribution of dI3 neurons transduced by AAV1-fl-sypGFP injections, plotted on an idealized sagittal section from T9 to L2 and colour coded per animal. (K) Correlation per animal between the median of the rostro-caudal distribution of transduced dI3 neurons somata in thoraco-lumbar segments and the median of the rostro-caudal position of their synaptic terminals mapped from segments C6 to T1, on the ipsilateral (left) and contralateral (right) side of the cord. Animals are colour coded as in panel J. (L) Spatial distribution (top) and density (bottom) of motor neurons innervating the caudal (green) and middle (magenta) part of the cutaneous maximus along the rostro-caudal axis on an idealized sagittal section from segments C6 to T1. (A) Spatial densities are pooled from tracing in n=3 mice per type of injection. (B-C) Data shown are from n=3 mice per type of injection. (G-H) n= 27 ipsilateral and n=21 contralateral cutaneous maximus motor neurons from N=3 mice. (I, L) Spatial distributions and densities are pooled from tracing in n=3 mice for dI3 terminals and n=4 mice for cutaneous maximus motor neurons. (K) R^2 and p values were calculated using the Spearman rank correlation coefficient.
